# (*E*)-3-(9-Hexyl-9*H*-carbazol-3-yl)acrylic acid

**DOI:** 10.1107/S1600536814016237

**Published:** 2014-07-31

**Authors:** Wan Sun, Wen-Mo Liu, Sheng-Li Li

**Affiliations:** a Department of Chemistry, Anhui University, Hefei 230039, People’s Republic of China; b Key Laboratory of Functional Inorganic Materials Chemistry, Hefei 230039, People’s Republic of China

**Keywords:** crystal structure

## Abstract

In the title compound, C_21_H_23_NO_2_, the hexyl group adopts an extended conformation, the six C atoms are nearly coplanar [maximum deviation = 0.082 (3) Å] and their mean plane is approximately perpendicular to the carbazole ring system, with a dihedral angle of 78.91 (15)°. In the crystal, mol­ecules are linked by O—H⋯O hydrogen bonds, forming inversion dimers; π–π stacking between carbazole ring systems of adjacent dimers further links the dimers into supra­molecular chains propagating along the *b*-axis direction [centroid-to-centroid distances = 3.868 (2) and 3.929 (2) Å].

## Related literature   

For structures of related carbazole derivatives, see: Saeed *et al.* (2010 ▶). For applications of carbazole derivatives, see: Adhikari *et al.* (2009 ▶); Daicho *et al.* (2013 ▶); Tao *et al.* (2010 ▶); Zheng *et al.* (2012 ▶); Dvornikov *et al.* (2009 ▶).
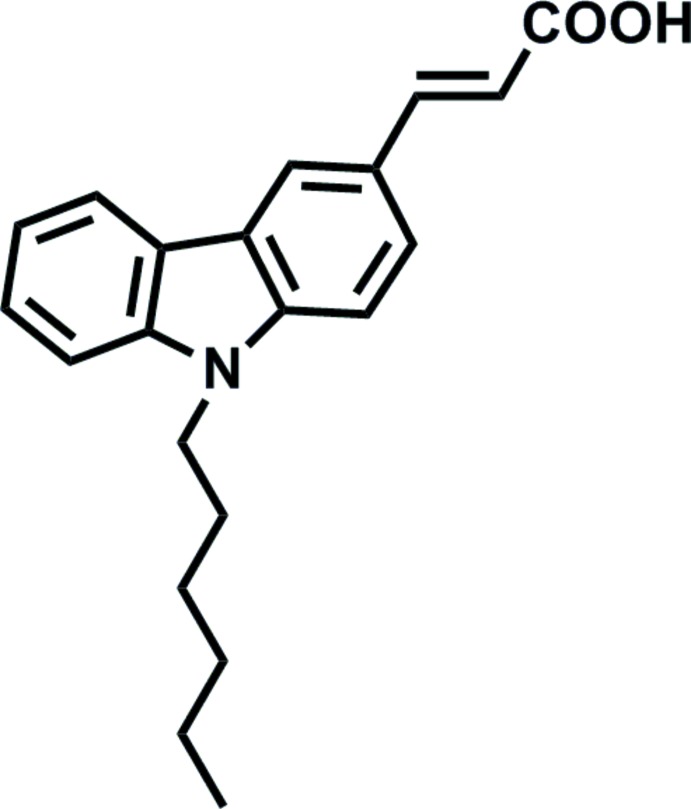


## Experimental   

### 

#### Crystal data  

C_21_H_23_NO_2_
*M* *_r_* = 321.40
Monoclinic, 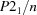

*a* = 10.594 (5) Å
*b* = 5.109 (2) Å
*c* = 33.152 (15) Å
β = 94.922 (6)°
*V* = 1787.5 (14) Å^3^
*Z* = 4
Mo *K*α radiation
μ = 0.08 mm^−1^
*T* = 298 K
0.30 × 0.20 × 0.20 mm

#### Data collection  

Bruker SMART APEX CCD diffractometer
11882 measured reflections
3115 independent reflections
2291 reflections with *I* > 2σ(*I*)
*R*_int_ = 0.027

#### Refinement  

*R*[*F* ^2^ > 2σ(*F* ^2^)] = 0.056
*wR*(*F* ^2^) = 0.192
*S* = 1.07
3115 reflections
219 parameters
H-atom parameters constrained
Δρ_max_ = 0.47 e Å^−3^
Δρ_min_ = −0.26 e Å^−3^

### 

Data collection: *SMART* (Bruker, 2007 ▶); cell refinement: *SAINT* (Bruker, 2007 ▶); data reduction: *SAINT*; program(s) used to solve structure: *SHELXTL* (Sheldrick, 2008 ▶); program(s) used to refine structure: *SHELXTL*; molecular graphics: *SHELXTL*; software used to prepare material for publication: *SHELXTL*.

## Supplementary Material

Crystal structure: contains datablock(s) I, Global. DOI: 10.1107/S1600536814016237/xu5796sup1.cif

Structure factors: contains datablock(s) I. DOI: 10.1107/S1600536814016237/xu5796Isup2.hkl

Supporting information file. DOI: 10.1107/S1600536814016237/xu5796Isup3.cml

CCDC reference: 992361

Additional supporting information:  crystallographic information; 3D view; checkCIF report

## Figures and Tables

**Table 1:** Hydrogen-bond geometry (Å, °)

*D*—H⋯*A*	*D*—H	H⋯*A*	*D*⋯*A*	*D*—H⋯*A*
O1—H1⋯O2^i^	0.82	1.85	2.650 (3)	166

Symmetry code: (i) 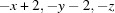.

**Fig. 1.**
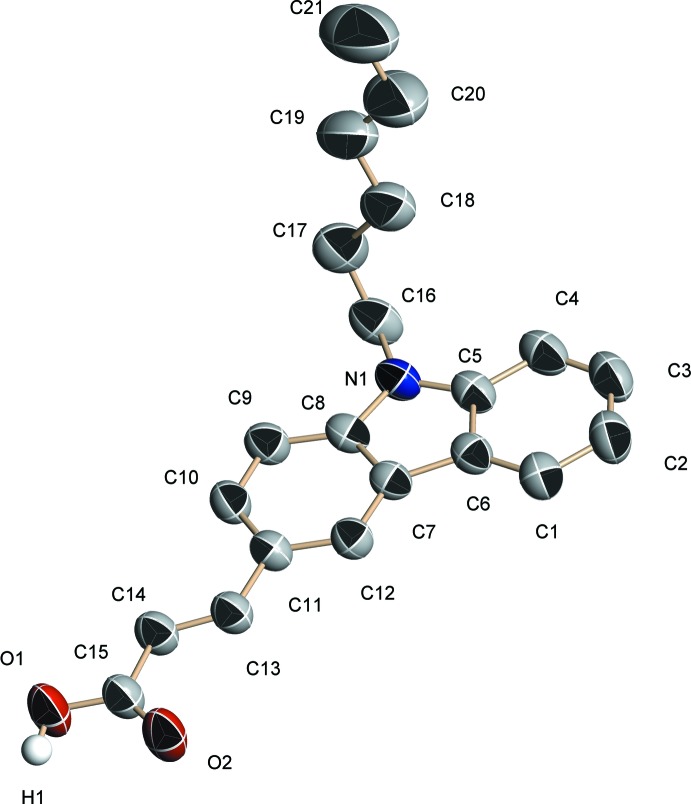
The molecular structure of the title compound showing 30% probability displacement ellipsoids.

## supplementary crystallographic information

### S1. Comment

Recently, carbazole derivatives have attracted attention as their superphotoelectric effect (Tao *et al.*, 2010) and electron transporting capabilities (Zheng *et al.*, 2012). So they have been widely used in biochemistry optical switching (Adhikari *et al.*, 2009), 3-D microfabrication (Daicho *et al.*, 2013) and optical data storage (Dvornikov *et al.*, 2009). In the present paper, the title carbazole derivative (Fig.1) is synthesized.

In the molecule, the carbazole and carboxylic acid are coplanar, while the hexyl group is nearly perpendicular to the plan of carbazole ring [dihedral angle = 78.91 (15)°]. The molecule connect with each other by intermolecular hydrogen-bonding O1—H1···O2.

### S2. Experimental

Carbazole single aldehyde (1.6 g, 5 mmol) and malonic acid (1.04 g, 10 mmol) was dissolved in pyridine with addition of 0.1 ml piperidine. The mixture was refluxed for 3 h, traced by TLC then column chromatography (silica, petroleum ether: ethyl acetate (V/V) = 5: 1) and finally 1.2 g white solid were acquired. Yield: 37%. 0.1 g LCOOH was dissolved in 30 ml me thanol, filtered to 50 ml volumetric flask, naturally evaporated for a week, and then colorless single crystals were obtained. ^1^H NMR (400 MHz, CD_3_COCD_3_) 0.84 (t, 3H), 1.33 (m, 6H), 1.89 (m, 2H), 4.46 (t, 2H), 6.59 (d, 1H), 7.26 (t, 1H), 7.50 (t, 1H), 7.62 (t, 2H), 7.82 (d, 1H), 7.87 (d, 1H), 8.23 (t, 1H), 8.51 (s, 1H), 10.53 (s, 1H).

### S3. Refinement

All hydrogen atoms were placed in geometrically idealized positions and constrained to ride on their parent atoms, with O—H = 0.82 and C—H = 0.93–0.97 Å, *U*_iso_(H) = 1.5*U*_eq_(C,O) for methyl H and hydroxyl H atoms, and 1.2*U*_eq_(C) for the others.

### Crystal data

**Table:**

C_21_H_23_NO_2_	*F*(000) = 688
*M**_r_* = 321.40	*D*_x_ = 1.194 Mg m^−^^3^
Monoclinic, *P*2_1_/*n*	Melting point: 425 K
Hall symbol: -P 2yn	Mo *K*α radiation, λ = 0.71073 Å
*a* = 10.594 (5) Å	Cell parameters from 2128 reflections
*b* = 5.109 (2) Å	θ = 4.2–20.6°
*c* = 33.152 (15) Å	µ = 0.08 mm^−^^1^
β = 94.922 (6)°	*T* = 298 K
*V* = 1787.5 (14) Å^3^	Block, yellow
*Z* = 4	0.30 × 0.20 × 0.20 mm

### Data collection

**Table:**

Bruker SMART APEX CCD diffractometer	2291 reflections with *I* > 2σ(*I*)
Radiation source: sealed tube	*R*_int_ = 0.027
Graphite monochromator	θ_max_ = 25.0°, θ_min_ = 1.2°
phi and ω scans	*h* = −12→12
11882 measured reflections	*k* = −6→5
3115 independent reflections	*l* = −39→38

### Refinement

**Table:**

Refinement on *F*^2^	Primary atom site location: structure-invariant direct methods
Least-squares matrix: full	Secondary atom site location: difference Fourier map
*R*[*F*^2^ > 2σ(*F*^2^)] = 0.056	Hydrogen site location: inferred from neighbouring sites
*wR*(*F*^2^) = 0.192	H-atom parameters constrained
*S* = 1.07	*w* = 1/[σ^2^(*F*_o_^2^) + (0.1029*P*)^2^ + 0.5377*P*] where *P* = (*F*_o_^2^ + 2*F*_c_^2^)/3
3115 reflections	(Δ/σ)_max_ < 0.001
219 parameters	Δρ_max_ = 0.47 e Å^−^^3^
0 restraints	Δρ_min_ = −0.26 e Å^−^^3^

### Special details

**Table:**

Geometry. All e.s.d.'s (except the e.s.d. in the dihedral angle between two l.s. planes) are estimated using the full covariance matrix. The cell e.s.d.'s are taken into account individually in the estimation of e.s.d.'s in distances, angles and torsion angles; correlations between e.s.d.'s in cell parameters are only used when they are defined by crystal symmetry. An approximate (isotropic) treatment of cell e.s.d.'s is used for estimating e.s.d.'s involving l.s. planes.
Refinement. Refinement of *F*^2^ against ALL reflections. The weighted *R*-factor *wR* and goodness of fit *S* are based on *F*^2^, conventional *R*-factors *R* are based on *F*, with *F* set to zero for negative *F*^2^. The threshold expression of *F*^2^ > σ(*F*^2^) is used only for calculating *R*-factors(gt) *etc*. and is not relevant to the choice of reflections for refinement. *R*-factors based on *F*^2^ are statistically about twice as large as those based on *F*, and *R*- factors based on ALL data will be even larger.

### Fractional atomic coordinates and isotropic or equivalent isotropic displacement parameters (Å^2^)

**Table:**

	*x*	*y*	*z*	*U*_iso_*/*U*_eq_	
C1	0.7326 (2)	0.3637 (5)	0.18010 (7)	0.0663 (7)	
H1A	0.8098	0.2762	0.1834	0.080*	
C2	0.7043 (3)	0.5596 (5)	0.20667 (8)	0.0774 (8)	
H2	0.7634	0.6047	0.2279	0.093*	
C3	0.5892 (3)	0.6895 (5)	0.20204 (8)	0.0758 (8)	
H3	0.5727	0.8210	0.2203	0.091*	
C4	0.4993 (3)	0.6291 (5)	0.17135 (8)	0.0698 (7)	
H4	0.4220	0.7164	0.1686	0.084*	
C5	0.5273 (2)	0.4319 (4)	0.14423 (7)	0.0570 (6)	
C6	0.6441 (2)	0.2999 (4)	0.14841 (6)	0.0535 (6)	
C7	0.64129 (19)	0.1119 (4)	0.11537 (6)	0.0500 (6)	
C8	0.5225 (2)	0.1420 (4)	0.09295 (6)	0.0526 (6)	
C9	0.4886 (2)	−0.0077 (5)	0.05871 (6)	0.0598 (6)	
H9	0.4102	0.0127	0.0442	0.072*	
C10	0.5756 (2)	−0.1879 (5)	0.04707 (6)	0.0587 (6)	
H10	0.5545	−0.2896	0.0242	0.070*	
C11	0.6947 (2)	−0.2234 (4)	0.06846 (6)	0.0529 (6)	
C12	0.7261 (2)	−0.0701 (4)	0.10286 (6)	0.0532 (6)	
H12	0.8045	−0.0909	0.1174	0.064*	
C13	0.7861 (2)	−0.4127 (4)	0.05570 (6)	0.0568 (6)	
H13	0.8624	−0.4229	0.0718	0.068*	
C14	0.7743 (2)	−0.5720 (5)	0.02407 (7)	0.0609 (6)	
H14	0.6993	−0.5668	0.0073	0.073*	
C15	0.8722 (2)	−0.7543 (5)	0.01418 (7)	0.0605 (6)	
C16	0.3276 (2)	0.4227 (5)	0.09634 (8)	0.0730 (8)	
H16A	0.3211	0.4295	0.0670	0.088*	
H16B	0.3152	0.5989	0.1062	0.088*	
C17	0.2239 (2)	0.2500 (7)	0.10958 (9)	0.0903 (10)	
H17A	0.1432	0.3267	0.0998	0.108*	
H17B	0.2298	0.0817	0.0963	0.108*	
C18	0.2226 (3)	0.2040 (7)	0.15327 (9)	0.0911 (9)	
H18A	0.2115	0.3705	0.1666	0.109*	
H18B	0.3042	0.1341	0.1635	0.109*	
C19	0.1198 (3)	0.0179 (7)	0.16456 (13)	0.1166 (13)	
H19A	0.1239	−0.1415	0.1488	0.140*	
H19B	0.0377	0.0976	0.1576	0.140*	
C20	0.1322 (5)	−0.0535 (9)	0.21078 (15)	0.1420 (17)	
H20A	0.2163	−0.1242	0.2177	0.170*	
H20B	0.1252	0.1065	0.2262	0.170*	
C21	0.0420 (5)	−0.2353 (11)	0.22313 (17)	0.182 (2)	
H21A	0.0430	−0.3896	0.2066	0.273*	
H21B	−0.0409	−0.1579	0.2201	0.273*	
H21C	0.0628	−0.2813	0.2510	0.273*	
N1	0.45361 (17)	0.3355 (4)	0.11062 (5)	0.0585 (5)	
O1	0.84699 (17)	−0.8937 (4)	−0.01813 (5)	0.0839 (6)	
H1	0.9106	−0.9745	−0.0233	0.126*	
O2	0.97385 (16)	−0.7732 (4)	0.03562 (5)	0.0849 (7)	

### Atomic displacement parameters (Å^2^)

**Table:**

	*U*^11^	*U*^22^	*U*^33^	*U*^12^	*U*^13^	*U*^23^
C1	0.0730 (16)	0.0645 (15)	0.0614 (14)	0.0008 (13)	0.0060 (12)	−0.0050 (12)
C2	0.095 (2)	0.0748 (19)	0.0621 (15)	−0.0061 (15)	0.0069 (13)	−0.0155 (13)
C3	0.099 (2)	0.0602 (16)	0.0706 (16)	0.0007 (15)	0.0238 (14)	−0.0148 (13)
C4	0.0798 (17)	0.0579 (15)	0.0750 (16)	0.0120 (13)	0.0253 (13)	−0.0025 (13)
C5	0.0688 (15)	0.0494 (13)	0.0548 (12)	0.0067 (11)	0.0171 (10)	0.0024 (10)
C6	0.0622 (13)	0.0493 (13)	0.0502 (12)	0.0028 (10)	0.0116 (10)	0.0018 (10)
C7	0.0546 (13)	0.0495 (13)	0.0470 (11)	0.0044 (10)	0.0108 (9)	0.0043 (9)
C8	0.0566 (13)	0.0546 (13)	0.0481 (11)	0.0076 (10)	0.0121 (9)	0.0044 (10)
C9	0.0546 (13)	0.0713 (15)	0.0532 (12)	0.0123 (12)	0.0036 (9)	0.0000 (11)
C10	0.0625 (14)	0.0648 (15)	0.0490 (12)	0.0049 (11)	0.0057 (10)	−0.0078 (10)
C11	0.0558 (13)	0.0536 (13)	0.0499 (12)	0.0084 (10)	0.0092 (9)	0.0006 (10)
C12	0.0539 (13)	0.0539 (14)	0.0519 (12)	0.0075 (10)	0.0040 (9)	0.0005 (10)
C13	0.0576 (13)	0.0588 (14)	0.0541 (12)	0.0078 (11)	0.0053 (10)	−0.0049 (11)
C14	0.0583 (14)	0.0676 (16)	0.0566 (13)	0.0130 (11)	0.0037 (10)	−0.0060 (11)
C15	0.0600 (14)	0.0662 (15)	0.0552 (13)	0.0116 (11)	0.0038 (10)	−0.0103 (11)
C16	0.0710 (17)	0.0803 (19)	0.0681 (15)	0.0281 (14)	0.0088 (12)	0.0062 (13)
C17	0.0615 (16)	0.123 (3)	0.0839 (19)	0.0204 (17)	−0.0066 (14)	−0.0058 (17)
C18	0.0790 (19)	0.089 (2)	0.107 (2)	0.0104 (16)	0.0196 (16)	−0.0084 (18)
C19	0.095 (2)	0.101 (3)	0.160 (3)	−0.012 (2)	0.052 (2)	−0.024 (2)
C20	0.151 (4)	0.126 (3)	0.158 (4)	−0.042 (3)	0.066 (3)	−0.020 (3)
C21	0.189 (5)	0.129 (4)	0.245 (6)	−0.028 (3)	0.117 (5)	−0.021 (4)
N1	0.0601 (11)	0.0592 (12)	0.0572 (11)	0.0153 (9)	0.0108 (9)	0.0002 (9)
O1	0.0744 (12)	0.1000 (15)	0.0750 (11)	0.0311 (11)	−0.0068 (9)	−0.0370 (10)
O2	0.0699 (12)	0.1028 (15)	0.0789 (12)	0.0305 (10)	−0.0121 (9)	−0.0354 (11)

### Geometric parameters (Å, º)

**Table:**

C1—C2	1.383 (4)	C14—C15	1.452 (3)
C1—C6	1.386 (3)	C14—H14	0.9300
C1—H1A	0.9300	C15—O2	1.242 (3)
C2—C3	1.385 (4)	C15—O1	1.295 (3)
C2—H2	0.9300	C16—N1	1.448 (3)
C3—C4	1.368 (3)	C16—C17	1.504 (4)
C3—H3	0.9300	C16—H16A	0.9700
C4—C5	1.399 (3)	C16—H16B	0.9700
C4—H4	0.9300	C17—C18	1.469 (4)
C5—N1	1.394 (3)	C17—H17A	0.9700
C5—C6	1.406 (3)	C17—H17B	0.9700
C6—C7	1.455 (3)	C18—C19	1.517 (5)
C7—C12	1.381 (3)	C18—H18A	0.9700
C7—C8	1.414 (3)	C18—H18B	0.9700
C8—N1	1.387 (3)	C19—C20	1.570 (6)
C8—C9	1.390 (3)	C19—H19A	0.9700
C9—C10	1.380 (3)	C19—H19B	0.9700
C9—H9	0.9300	C20—C21	1.418 (6)
C10—C11	1.405 (3)	C20—H20A	0.9700
C10—H10	0.9300	C20—H20B	0.9700
C11—C12	1.400 (3)	C21—H21A	0.9600
C11—C13	1.457 (3)	C21—H21B	0.9600
C12—H12	0.9300	C21—H21C	0.9600
C13—C14	1.325 (3)	O1—H1	0.8200
C13—H13	0.9300		
			
C2—C1—C6	118.9 (2)	O2—C15—C14	121.4 (2)
C2—C1—H1A	120.5	O1—C15—C14	116.04 (19)
C6—C1—H1A	120.5	N1—C16—C17	113.6 (2)
C1—C2—C3	120.9 (2)	N1—C16—H16A	108.8
C1—C2—H2	119.6	C17—C16—H16A	108.8
C3—C2—H2	119.6	N1—C16—H16B	108.8
C4—C3—C2	121.6 (2)	C17—C16—H16B	108.8
C4—C3—H3	119.2	H16A—C16—H16B	107.7
C2—C3—H3	119.2	C18—C17—C16	116.8 (2)
C3—C4—C5	117.9 (2)	C18—C17—H17A	108.1
C3—C4—H4	121.1	C16—C17—H17A	108.1
C5—C4—H4	121.1	C18—C17—H17B	108.1
N1—C5—C4	129.3 (2)	C16—C17—H17B	108.1
N1—C5—C6	109.70 (19)	H17A—C17—H17B	107.3
C4—C5—C6	121.0 (2)	C17—C18—C19	114.3 (3)
C1—C6—C5	119.7 (2)	C17—C18—H18A	108.7
C1—C6—C7	134.0 (2)	C19—C18—H18A	108.7
C5—C6—C7	106.33 (18)	C17—C18—H18B	108.7
C12—C7—C8	119.20 (19)	C19—C18—H18B	108.7
C12—C7—C6	134.30 (19)	H18A—C18—H18B	107.6
C8—C7—C6	106.49 (18)	C18—C19—C20	112.6 (3)
N1—C8—C9	128.9 (2)	C18—C19—H19A	109.1
N1—C8—C7	109.38 (19)	C20—C19—H19A	109.1
C9—C8—C7	121.7 (2)	C18—C19—H19B	109.1
C10—C9—C8	117.6 (2)	C20—C19—H19B	109.1
C10—C9—H9	121.2	H19A—C19—H19B	107.8
C8—C9—H9	121.2	C21—C20—C19	115.6 (4)
C9—C10—C11	122.5 (2)	C21—C20—H20A	108.4
C9—C10—H10	118.8	C19—C20—H20A	108.4
C11—C10—H10	118.8	C21—C20—H20B	108.4
C12—C11—C10	118.63 (19)	C19—C20—H20B	108.4
C12—C11—C13	119.4 (2)	H20A—C20—H20B	107.4
C10—C11—C13	122.0 (2)	C20—C21—H21A	109.5
C7—C12—C11	120.40 (19)	C20—C21—H21B	109.5
C7—C12—H12	119.8	H21A—C21—H21B	109.5
C11—C12—H12	119.8	C20—C21—H21C	109.5
C14—C13—C11	128.1 (2)	H21A—C21—H21C	109.5
C14—C13—H13	115.9	H21B—C21—H21C	109.5
C11—C13—H13	115.9	C8—N1—C5	108.09 (18)
C13—C14—C15	123.5 (2)	C8—N1—C16	125.8 (2)
C13—C14—H14	118.3	C5—N1—C16	126.14 (19)
C15—C14—H14	118.3	C15—O1—H1	109.5
O2—C15—O1	122.5 (2)		
			
C1—C6—C7—C12	−0.7 (5)	N1—C5—C4—C3	−179.6 (2)
C5—C6—C7—C12	179.3 (2)	C6—C5—C4—C3	0.2 (4)
C1—C6—C7—C8	180.0 (3)	C15—C14—C13—C11	180.0 (2)
C5—C6—C7—C8	0.0 (2)	C12—C11—C13—C14	−179.3 (3)
C8—C7—C12—C11	0.0 (3)	C10—C11—C13—C14	0.2 (4)
C6—C7—C12—C11	−179.3 (2)	N1—C8—C9—C10	179.5 (2)
C10—C11—C12—C7	−0.1 (3)	C7—C8—C9—C10	−0.2 (4)
C13—C11—C12—C7	179.5 (2)	C8—C9—C10—C11	0.1 (4)
C5—N1—C8—C9	−179.5 (2)	C12—C11—C10—C9	0.1 (4)
C16—N1—C8—C9	−0.1 (4)	C13—C11—C10—C9	−179.5 (2)
C5—N1—C8—C7	0.3 (3)	C5—C4—C3—C2	−0.5 (4)
C16—N1—C8—C7	179.7 (2)	C13—C14—C15—O2	1.2 (4)
C12—C7—C8—N1	−179.60 (19)	C13—C14—C15—O1	−178.9 (3)
C6—C7—C8—N1	−0.2 (2)	C5—C6—C1—C2	−0.6 (4)
C12—C7—C8—C9	0.2 (3)	C7—C6—C1—C2	179.3 (3)
C6—C7—C8—C9	179.6 (2)	C6—C1—C2—C3	0.3 (4)
C8—N1—C5—C4	179.4 (2)	C4—C3—C2—C1	0.3 (5)
C16—N1—C5—C4	0.1 (4)	C16—C17—C18—C19	−177.5 (3)
C8—N1—C5—C6	−0.4 (3)	C17—C18—C19—C20	173.0 (3)
C16—N1—C5—C6	−179.7 (2)	C18—C19—C20—C21	−177.3 (4)
C1—C6—C5—N1	−179.8 (2)	C8—N1—C16—C17	81.9 (3)
C7—C6—C5—N1	0.2 (3)	C5—N1—C16—C17	−98.9 (3)
C1—C6—C5—C4	0.4 (4)	C18—C17—C16—N1	55.5 (4)
C7—C6—C5—C4	−179.6 (2)		

### Hydrogen-bond geometry (Å, º)

**Table:**

*D*—H···*A*	*D*—H	H···*A*	*D*···*A*	*D*—H···*A*
O1—H1···O2^i^	0.82	1.85	2.650 (3)	166

Symmetry code: (i) −*x*+2, −*y*−2, −*z*.

## References

[bb1] Adhikari, R. M., Shah, B. M., Palayangoda, S. S. & Neckers, D. C. (2009). *Langmuir*, **25**, 2402–2406. 10.1021/la802716w 19154136

[bb2] Bruker (2007). *SAMART* and *SAINT* Bruker AXS Inc., Madison, Wisconsin, USA.

[bb3] Daicho, Y., Murakami, T., Hagiwara, T. & Maruo, S. (2013). *Opt. Mater. Express*, **3**, 873–883.

[bb4] Dvornikov, A. S., Walker, E. P. & Rentzepis, P. M. (2009). *J. Phys. Chem. A*, **113**, 13633–13644. 10.1021/jp905655z 19856937

[bb5] Saeed, A., Kazmi, M., Ameen Samra, S., Irfan, M. & Bolte, M. (2010). *Acta Cryst.* E**66**, o2118. 10.1107/S1600536810028928 PMC3007423 21588408

[bb6] Sheldrick, G. M. (2008). *Acta Cryst.* A**64**, 112–122. 10.1107/S0108767307043930 18156677

[bb7] Tao, Y.-T., Wang, Q., Yang, C.-L., Cheng, Z. & Ma, D.-G. (2010). *Adv. Funct. Mater.* **20**, 304–311.

[bb8] Zheng, C.-J., Ye, J. & Lo, M.-F. (2012). *Chem. Mater.* **24**, 643–650.

